# Tumor-Mediated Neutrophil Polarization and Therapeutic Implications

**DOI:** 10.3390/ijms23063218

**Published:** 2022-03-16

**Authors:** Sofia Raftopoulou, Paulina Valadez-Cosmes, Zala Nikita Mihalic, Rudolf Schicho, Julia Kargl

**Affiliations:** Division of Pharmacology, Otto Loewi Research Center, Medical University of Graz, 8010 Graz, Austria; sofia.raftopoulou@medunigraz.at (S.R.); paulina.valadez-cosmes@medunigraz.at (P.V.-C.); zala.mihalic@medunigraz.at (Z.N.M.); rudolf.schicho@medunigraz.at (R.S.)

**Keywords:** tumor microenvironment, immune cells, neutrophils

## Abstract

Neutrophils are immune cells with reported phenotypic and functional plasticity. Tumor-associated neutrophils display many roles during cancer progression. Several tumor microenvironment (TME)-derived factors orchestrate neutrophil release from the bone marrow, recruitment and functional polarization, while simultaneously neutrophils are active stimulators of the TME by secreting factors that affect immune interactions and subsequently tumor progression. Successful immunotherapies for many cancer types and stages depend on the targeting of tumor-infiltrating lymphocytes. Neutrophils impact the success of immunotherapies, such as immune checkpoint blockade therapies, by displaying lymphocyte suppressive properties. The identification and characterization of distinct neutrophil subpopulations or polarization states with pro- and antitumor phenotypes and the identification of the major TME-derived factors of neutrophil polarization would allow us to harness the full potential of neutrophils as complementary targets in anticancer precision therapies.

## 1. Cancer and the Tumor Microenvironment

Our knowledge of the relationship between the immune system and cancer dates back to the 19th century when Rudolf Virchow reported the presence of leukocytes in neoplastic tissues [1]. Currently, the study of tumor-associated immune cells constitutes a central element within the research community and our understanding of cancer has been (re)shaped by understanding the tumor microenvironment (TME). The TME comprises a complex and unique network of proliferating tumor cells, blood vessels, extracellular matrix components, and non-neoplastic cells, which includes stromal, endothelial and infiltrated inflammatory cells [2]. Tumor cells secrete a broad range of cytokines and chemokines that not only regulate the behavior of stromal and tumor cells themselves but also attract and affect diverse immune cells. Once recruited into the tumor, infiltrated immune cells will also influence the recruitment, activation and behavior of other leukocytes, as reviewed by Binnewies et al. [3]. The integration of this sophisticated network has helped us understand the diverse and sometimes conflicting roles of specific immune cell populations in the setting of cancer. Furthermore, the development of more sophisticated techniques and the emergence of new cellular markers have allowed the identification of new tumor-associated immune cell subpopulations, highlighting the heterogeneity and complexity of the immune landscape in cancer. The research efforts have been focused not only on understanding the role of immune cells in tumor fate but also on their potential use as diagnostic tools and for the improvement of immune-based cancer therapies.

One of the immune cell populations that have become increasingly interesting in the setting of cancer are neutrophils. Current research has shown that neutrophils can oppose or enhance cancer progression [4]. Such contrary roles may be caused by the influence that the TME has on recruiting and promoting neutrophils with specific phenotypes and functions. In this review, we provide an overview of the different neutrophil subsets and their roles in cancer. We highlight the TME-derived factors that drive neutrophil polarization and infiltration as well as the neutrophil-derived factors and the feedback loops that occur within the TME. Finally, we discuss the current strategies and limitations of targeting neutrophils in the TME.

## 2. Neutrophil Development and Physiological Role

Neutrophils are short-lived cells representing the majority (50–70%) of all immune cells in the peripheral blood; furthermore, increased neutrophil numbers have been shown in various cancers [4]. Upon inflammation, neutrophils are the first cells that respond and are rapidly recruited to the site of inflammation, where they act to eliminate infections [5]. The development of neutrophils starts from self-renewing hematopoietic stem cells in the bone marrow, during a process called granulopoiesis. They differentiate into multipotent hematopoietic progenitors, which are developed into common myeloid progenitors and common lymphoid progenitors. On the way towards mature neutrophils, common myeloid progenitors evolve to granulocyte or monocyte progenitors [6]. The differentiation of progenitor cells is controlled by granulopoiesis stimulating factors, such as granulocyte colony-stimulating factor (G-CSF), which regulates the formation of myeloblasts, and their evolvement towards mature neutrophils through the stages of promyelocyte, myelocyte, metamyelocyte, and band cell, respectively [7]. Furthermore, G-CSF downregulates CXCL12 and its receptor CXCR4. Their interaction results in the retention of neutrophils in the bone marrow [8].

To resolve inflammation, tissue-resident macrophages and mast cells secrete several chemokines and cytokines, which activate endothelial cells to express adhesion molecules such as P- and E-selectin [9]. This causes the rolling of neutrophils along the endothelium. Furthermore, neutrophils are activated by chemokines, and they express β2 integrins, resulting in firm binding of neutrophils to integrin ligands, such as intracellular adhesion molecules 1 and 2. Upon firm adhesion, neutrophils transmigrate through the endothelial cell layer to the site of inflammation and along the chemokine gradient, toward the center of infection [10,11]. As Lehman and Segal recently reviewed [12], neutrophils can resolve inflammation through different mechanisms: pathogen recognition and phagocytosis, degranulation, oxidative burst and formation of neutrophil extracellular traps (NETs). Finally, neutrophils are removed from the tissue by macrophage phagocytosis [13]. Additional to the role of neutrophils in resolving acute infections, neutrophils affect chronic inflammatory conditions such as autoimmune diseases and cancers [14]. Furthermore, it has been shown, that upon increased demand, emergency granulopoiesis can be induced by the hematopoietic system [15]. It has been suggested that neutrophils do not just eliminate microbes, but also localize in some organs, such as the spleen, liver, and lung, where they interact with other immune cells and modulate their function [16].

## 3. The Opposing Roles of Neutrophils in Cancer

The recruitment of immune cells to the tumor site portrays the immune system’s battle to resolve the persistent cancer-related inflammation, which promotes tumor initiation and progression and supports the avoidance of immunosurveillance. The presence of myeloid cells in the complex landscape of the TME holds pivotal roles. From all tumor-infiltrating myeloid cells, macrophages are the most extensively studied and best characterized cells in terms of diversity and functionality. However, knowledge on tumor-infiltrating neutrophils lags behind, as they have only recently come to the spotlight. Neutrophils represent a highly heterogeneous population, capable of both anti- and protumor functions, which is most attributable to phenotypic plasticity.

### 3.1. Protumorigenic Functions of Neutrophils

Inflammation holds a key role in initiating tumorigenesis, with neutrophils being a distinct component of this process. The exact contribution of neutrophils to tumorigenesis is not yet fully understood. Neutrophil-derived enzymes such as neutrophil elastase (NE) [17,18], the production of reactive oxygen species (ROS) [19,20] and reactive nitrogen species (RNS), as well as the immunosuppressive ability of a subset of neutrophils have all been reported to be implicated in tumor initiation. A tumor promoting role has also been attributed to neutrophils by in vivo studies. One main neutrophil-controlled mechanism proposed to assist in the promotion of tumor growth is the induction of angiogenesis [21]. Neutrophil regulated immunosuppression is considered another major mechanism that facilitates tumor progression [22]. Finally, the release of extracellular neutrophil-derived networks of DNA, fibers and proteins (NETs) is yet another mechanism that facilitates tumor progression [23]. Many studies indicate that neutrophils are important components during the initiation of metastasis. Intravital imaging has revealed co-localization of cancer cells with endothelial cell-associated neutrophils, suggesting that neutrophils guide cancer cells into tissues and/or retain them there [24]. NETs can sequester circulating cancer cells and promote their adhesion at distant organs [25]. A recent study by Aceto et al. showed that neutrophils cluster with circulating tumor cells (CTCs) in the peripheral blood of patients with breast cancer and in mouse models. The formation of CTC–neutrophil clusters drives cell cycle progression within the bloodstream and expands the metastatic potential of CTCs [26]. Finally, regarding the clinical implications, the neutrophil to lymphocyte ratio (NLR) has been proposed as a biomarker for risk stratification of patients with cancer [27,28]. A rise in neutrophil counts and/or NLR indicates disease recurrence or progression, and a drop in the NLR after initiation of therapy indicates a good response [29]. More recently, a study showed that the ratio of CD8^+^ T cells to neutrophils within the tumor could separate patients responsive to anti-PD-1 therapy from those with stable or progressive disease in non-small cell lung cancer (NSCLC) [30].

### 3.2. Antitumorigenic Functions of Neutrophils

Neutrophils facilitate their phagocytic function through the production of various antimicrobial molecules. Among them, superoxides H_2_O_2_ and HOCl [31] are directly involved in antitumor cytotoxicity [32]. Although several studies have shown that physical contact is required for neutrophil cytotoxicity, stimulating cultured neutrophils with phorbol myristate acetate (PMA) led to the generation and secretion of high levels of H_2_O_2_, bypassing the necessity of physical contact [33]. Antibody-dependent cell-mediated cytotoxicity (ADCC) is another cytotoxic mechanism used by neutrophils through the expression of Fc receptors that mediate ADCC in several types of cancers, including Non-Hodgkin lymphoma, breast cancer and B cell lymphoma [34,35,36]. Recently, PD-1-expressing tumor cells inhibited neutrophil cytotoxicity via the PD-L1/PD-1 axis [37]. Neutrophils also exert indirect antitumor effects since they can also stimulate adaptive antitumor immune responses both by facilitating the recruitment of other immune cells [38] and by possessing an antigen-presenting potential on their own [39,40]. Neutrophils can recruit and activate T cells by secreting cytokines, such as tumor necrosis factor alpha (TNFα), and Cathepsin G promotes T cell proliferation and cytokine production [41]. Eruslanov et al. proposed that tumor-associated neutrophils (TANs) can stimulate T cell proliferation and IFN-γ secretion in early-stage lung cancer patients [42]. NETs have also been reported to be used for T cell priming and to present a supportive function to the antitumor immune responses [43]. A recent study proposed that the outcome of neutrophil–T cell contact can depend on the activation status of both cell types [44]. Finally, the ability of antigen presentation to T cells, which was thought to be exclusively owned by macrophages and dendritic cells (DCs), has also been attributed to antigen-presenting cell (APC)-like neutrophils according to studies conducted over the past decade [45,46]. Although these studies propose an antitumorigenic role of neutrophils, attributed to T cell stimulatory effects, most studies so far suggest that neutrophils predominantly exert immunosuppressive functions [22].

## 4. Neutrophil Secreted Factors That Shape Immune Cell Interactions within the TME

The function of many immune cell populations, including macrophages, DCs, T cells, and NK cells, is modulated within and by the TME. For example, tumor-associated macrophages (TAMs), which represent a cytotoxic inflammatory phenotype (M1) at the early stages of tumor development, are polarized toward a protumor, proangiogenic and immunosuppressive phenotype (M2) during disease progression [47]. Tumor-infiltrating DCs and NK cells are suppressed in the TME, displaying similar polarization patterns [48,49]. Suppression and exclusion of cytotoxic CD8^+^ T cells is another mechanism that inhibits the antitumor immune responses [50]. Cytotoxic T lymphocyte (CTL) exclusion can be provoked by several mechanisms, such as the blocking of CTL recruitment, inhibition of T cell functions through the PD-L1 checkpoint and changes in the extracellular matrix that block T cell infiltration. Moreover, T cell subsets with protumor properties such as CD4^+^ Tregs are attracted over time to the TME [51].

**Figure 1:**
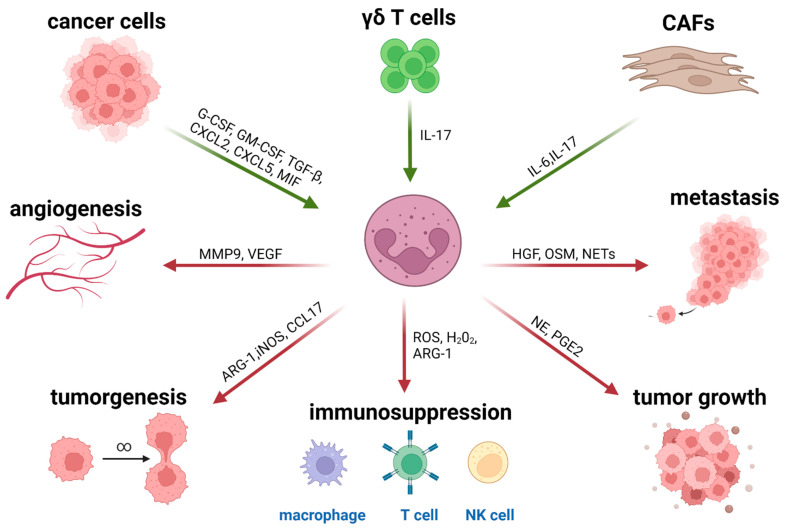
Neutrophils are recruited to the tumor site in response to TME-derived stimuli such as chemokines and cytokines (green arrows). Polarized TANs exert immunosuppressive and protumor functions with the aid of neutrophil-derived factors such as granule enzymes and reactive oxygen species (red arrows).

Neutrophils recruited into the TME (tumor-associated neutrophils, TANs) are a source of cytokine and chemokine secretion that impacts innate and adaptive immunity as portrayed in Figure 1. TANs secrete cytokines and chemotactic cytokines (referred to as chemokines), which can control their own recruitment as well as the recruitment and activation of other immune cells [52]. TANs are a heterogeneous population [53] that can support anti or/and protumor functions. TANs possess an antitumor phenotype, and express high levels of proinflammatory cytokines such as IL-12 and TNF-α. Additionally, proinflammatory TANs secrete several chemokines (CXCL10, CCL7, CCL2 and CCL3) that serve as T cell and macrophage chemo-attractants. However, TANs of a protumor phenotype express chemokines such as CCL17 and CXCL14. Secretion of CCL17 by TANs was shown to be followed by an active recruitment of Tregs to the TME, which was impaired after TAN depletion, while CXCL14 attracts activated macrophages, immature DCs, and NK cells [54]. In contrast, in mouse studies, CCL17 was also shown to recruit antitumor leukocytes to the tumor site [55]. Transcriptome analysis by Fridlender et al. also indicated that the expression of Transforming growth factor beta (TGF-β1), IL-6 and IL-23 by anti-inflammatory TANs may promote Th17 priming [56]. An extensive network of cross-talks connects TANs to other tumor-infiltrating immune cells. TANs can promote CD8^+^ T cell recruitment and activation through the production of T cell chemo-attractants (e.g., CCL3, CXCL9, and CXCL10) and proinflammatory cytokines (IL-12, TNF-α and GM-CSF). Additionally, TANs can activate DCs via cell–cell contact and secretion of TNF-α [57] as well as facilitate metastasis by inhibiting NK activation and IFN-γ secretion [58]. Finally, TANs attract and activate macrophages by secreting IL-8, TNF-α, and myeloperoxidase (MPO) [59]. MPO, an enzyme abundantly expressed in neutrophil granulocytes, is upregulated and secreted by TANs in several cancer types [60]. MPO is involved in early stages of cancer development [61] and has been proposed to influence the TAN–TAM axis [62,63].

## 5. Neutrophil Recruitment into the TME

The neutrophil life cycle starts in the bone marrow where premature neutrophils are derived from hematopoietic stem cells. The mobilization and release of neutrophils from the bone marrow into the circulation and their recruitment at the tumor sites is a multistep process strictly guided by several factors. Two G-protein coupled receptors of the CXC chemokine receptor family and their corresponding ligands are mainly responsible for the release of neutrophils from the bone marrow. CXCR4 and CXCR2 are expressed on the surface of neutrophils [64]. CXCR4 serves for neutrophil homing in the bone marrow. High expression levels of CXCR4 and its ligands, such as CXCL12, result in restriction of neutrophil mobility. The disruption of the expression of CXCR4 and its ligands by factors such G-CSF results in the initiation of neutrophil mobilization. In contrast, CXCR2 and its ligands, in coordination with G-CSF, are mainly responsible for the release of neutrophils into the circulation. The maintenance of neutrophil homeostasis in circulation is based on the antagonistic interaction between CXCR2 and CXCR4 [65,66]. Upregulation of CXCR2 expression determines the mobilization of mature neutrophils into the circulation, while the upregulation of CXCR4 on aged neutrophils leads to their regression into the bone marrow where they end up being digested by macrophages [67]. In the setting of cancer, the CXCR2 axis plays a dominate role in neutrophil recruitment into the TME. The occurrence of a solid tumor and the consequently required mobilization of neutrophils to the tumor site depends on an axis of interactions among CXCR2 and its ligands CXCL1-3 and CXCL5-8 [68,69]. Once there is a need for neutrophil mobilization, chemokines for CXCR2 need to be released into the circulation. Among the cells that shape the TME, tumor cells, immune cells, and cancer-associated fibroblasts are the main producers of CXCR2 chemokines. Neutrophils respond to this chemokine release by moving through a chemotactic gradient, toward the higher concentration of CXCR2 ligands. For this chemotactic process to occur, both the expression of CXCR2 on the neutrophil surface and the production of CXCR2 ligands are essential [70].

As previously mentioned, G-CSF is a potent regulator of neutrophil recruitment, while also being implicated in neutrophil proliferation, maturation and function. G-CSF is a cytokine produced by several cells, including macrophages, endothelial cells and cancer cells [71]. G-CSF positively regulates neutrophil migration by provoking a decrease in the expression of CXCR4 and its ligand, CXCL12. Blocking of the G-CSF receptor in mice led to inhibition of neutrophil mobilization [72]. However, G-CSF does not directly induce neutrophil chemotaxis, but indirectly and mainly through interactions among CXCR4, CXCR2 and ligands. Members of the CXCL chemokine family are potent chemotactic factors for neutrophils. Human CXCL8 (IL-8) is produced by stromal and tumor cells and is one of the best studied neutrophil chemo-attractants in cancer. IL-8 is overexpressed in several carcinomas and tumor cell lines including breast, colon, cervical, lung, brain, prostate, ovarian, renal cell carcinomas, acute myelogenous, B cell lymphocytic leukemia, melanoma and Hodgkin’s disease [73]. Interleukin-17 (IL-17) is another key component of neutrophil recruitment in the TME [74]. The IL-17 family consists of six members, named IL-17A–F. IL-17A, simply known as IL-17, shows a positive correlation with neutrophil counts in the TME and was found to upregulate the expression of G-CSF, IL6, CCL2 (MCP-1), and CXCR2 ligands [75]. IL-17 increased the secretion of CXCL1 and CXCL5 by carcinoma cells in breast cancer models, facilitating cancer progression. Consequently, higher levels of IL-17 in breast cancer patients have been correlated with lower survival rates [76]. Neutrophil recruitment can also be facilitated by additional factors; the hypoxia-inducible factor 1-α (HIF1- α) and its downstream products, such as CXCL12, vascular endothelial growth factor (VEGF), or matrix metallopeptidase 9 (MMP9) are involved in the recruitment and retention of neutrophils during angiogenesis [77]. In particular, VEGF, when highly expressed, can induce neutrophil adhesion and homing toward the tumor or the premetastatic niche [78]. Sphingosine-1-phosphate (S1P), a bioactive lipid, also promotes neutrophil activation and chemotaxis [79]. Finally, myeloid-related proteins (MRPs) are also involved in neutrophil migration, although the exact mechanism is not clear yet. The MRPs S100A8 and S100A9 are highly expressed in the TME and the premetastatic niche, serving neutrophil recruitment [80].

## 6. Neutrophil Phenotypic Polarization Is Orchestrated by the TME

### 6.1. Pro- and Antitumor TAN Phenotypes

Heterogeneity and functional plasticity are common characteristics of leukocytes. Macrophages, for example, used to be subcategorized based on their activation status, in the classically activated, proinflammatory M1 type and alternatively activated, anti-inflammatory or immunosuppressive M2 type [81], with additional subtypes more recently defined. TAMs potentially become polarized from an antitumor (M1) to a protumor (M2) activation mode by the highly active and complex secretome of the TME [82]. Similar to TAMs, neutrophils have recently been proposed to exhibit degrees of phenotypic plasticity. The existence of different neutrophil subsets concerning their maturation and activation status as well as their pro- and antitumor functions comes in contrast to the previous knowledge that mature neutrophils leave the bone marrow as terminally differentiated cells. Thus far, neutrophil subsets with opposite functions have been identified in the circulation and primary tumors of cancer patients [83]. Additionally, studies in mouse tumor models identified the presence of tumor-associated neutrophils with different activation status. Following the TAM paradigm, these TANs were proposed to be described as antitumor N1 and protumor N2. TGF-β, which is overexpressed in many tumors, was proposed by Fridlender et al. as a key stimulator of TAN polarization between the N1 and N2 phenotypes in murine mesothelioma and lung cancer models. Inhibition of TGF-β enhanced the proinflammatory potential of TANs (N1), including cytotoxic CD8+ T lymphocyte activation, reactive oxygen species-dependent direct killing of tumor cells, and high expression of proinflammatory cytokines such as TNF-α and CCL3 and the costimulatory molecule ICAM-1. Simultaneously, low expression of arginase (ARG-1), an immunosuppressive enzyme, was observed [46]. In another mouse study using tumor-bearing IFN-β ^−/−^ mice, TANs displayed protumor behavior, such as reduced expression of ICAM-1 and TNF-α and low cytotoxicity toward tumor cells, implying that IFN-β enhances the polarization of TANs toward the antitumor phenotype [84].

The effect of TME-derived soluble factors on neutrophil polarization is not restricted to the tumor site. Tumor-derived G-CSF was identified by Casbon et al. and plays a major role in the reprogramming of myeloid differentiation in the bone marrow. This reprogramming results in the expansion of T cell suppressive neutrophils in the peripheral tissues during tumorigenesis in an oncogene-driven murine breast cancer model [71]. The role of these T cell suppressive neutrophils during tumor progression and metastasis needs to be addressed in further studies. Thus far, there are no conclusive data regarding the extent of the influence that the TME secretome has on TAN phenotype polarization. Whether these contradictory phenotypes appear on distinct TAN subpopulations or whether these TANs stretch across a reversible activation status scale remains to be investigated. For instance, Pfirschke et al. recently showed in mice that tumor-infiltrating CD11b^+^Ly6G^+^SiglecF^high^ cells are bona fide mature neutrophils, different from other SiglecF-expressing myeloid cells [85]. In humans, similar TAN phenotypes have yet to be characterized and functionally tested.

### 6.2. Immature Neutrophils and G-MDSCs

Fully mature neutrophils are morphologically typically characterized by segmented nuclei. Oppositely, immature neutrophil subsets present a banded, ring-shaped or non-segmented nuclear morphology. Immature neutrophils with a ring-like nuclear morphology were detected in the blood of tumor-bearing IFN-β ^−/−^ mice [84]. Immature subsets also coincided with a protumor TAN phenotype described in the study of Fridlender et al. [46]. Expansion of immature neutrophils was more recently observed in the circulation, primary tumors, and distant organs of mammary tumor-bearing mice. Those neutrophils suppressed CD8^+^ T cell proliferation and activation, resulting in enhanced metastasis formation [86]. Myeloid-derived suppressor cells represent a non-lymphoid immune suppressor cell population of myeloid origin, enriched in cancer patients [87]. MDSCs constitute a population of myeloid cells with heterogeneous morphology, surface phenotype, and function, but with common strong immunosuppressive properties. MDSCs play an important role in regulating immune responses, mostly by suppressing T cell responses [88]. MDSCs have also been described to regulate innate immune responses by modulating the cytokine production of macrophages [89]. In terms of morphology, surface phenotype, and function, MDSCs are not a defined subset, but rather a heterogeneous population. They express a mixture of surface markers typical for myeloid cells, but lack lineage markers for lymphocytes, natural killer cells, macrophages, and dendritic cells [90]. Two major groups of MDSCs have been characterized so far: those with morphology and surface phenotype typical of monocytes (M-MDSCs) and those with a surface phenotype typical of granulocytes (G-MDSCs—also called polymorphonuclear (PMN)-MDSCs), but with a heterogeneous morphology including granulocytes, blasts, or cells with ring-shaped nuclei [91]. There is a debate supported by several studies that M-MDSCs and G-MDSCs represent monocytes and neutrophils, respectively, that have been reprogrammed or activated into immunosuppressive populations [92]. Myeloid-derived suppressor cells of granulocytic origin (G-MDSCs) expand in the spleen of tumor-bearing mice and migrate to the TME where they characteristically suppress cytotoxic T cell responses [93]. Seemingly, immature neutrophil subsets possess protumor functions comparable to those of G-MDSCs since both populations share common surface markers and morphologic features. The possibility, however, that they represent the same functional subset of neutrophils remains controversial. Transcriptomic profiling in tumor-bearing mice revealed that TANs represent a distinct population from splenic G-MDSCs, without necessarily excluding the fact that G-MDSCs may be converted into TANs under the influence of the TME [94]. The abundance of mature neutrophils in primary tumors of 4T1 breast tumor mouse models was influenced by host CCL5, produced by myeloid cells in a CCR5-CCL5 autocrine manner, rather than by tumor-derived CCL5, resulting in the generation of immunosuppressive immature Ly6G+ myeloid cells. When host production of CCL5 was blocked, neutrophils recruited into the TME represented an antitumor phenotype [95]. Most studies highlight the fact that neutrophil phenotypic plasticity is present during tumorigenesis and tumor progression, coexisting with changes on their maturation status, an effect primarily but not exclusively orchestrated by the TME. Marini et al. identified CD10 as a cell surface marker that distinguishes T cell suppressive from T cell stimulatory neutrophils in the peripheral blood of cancer and systemic lupus erythematosus (SLE) patients [96]. In the same study, immunosuppressive mature CD66b^+^CD10^+^ and immunostimulatory immature CD66b^+^CD10^−^ neutrophils coexist in G-CSF-treated donors, a finding that has major implications for our understanding of how neutrophils are modulated by factors derived by the TME.

### 6.3. High- vs. Low-Density Neutrophils

The presence of neutrophils of heterogeneous maturation status in the circulation has been documented in cancer patients. A mixed population of immature neutrophils with ring- or band-shaped nuclei and mature neutrophils with segmented nuclei [97], addressed as low-density neutrophils (LDNs), have been reported during disease progression in the peripheral blood of patients with head and neck, lung, and urologic cancers [98]. LDNs were also identified by Sagiv et al. in the blood of patients with advanced-stage lung and breast cancer, as well as in mouse models of breast, mesothelioma, and lung cancer [97]. LDNs occur in the low-density mononuclear fraction during density gradient centrifugation of blood, in contrast to normal granulocytes that have a high density. LDNs also represent different maturation stages of neutrophils, as portrayed by their distinct nuclear morphology and differential expression of surface markers [99]. In human cancers, LDNs overexpress CD66b, CD11b and CD15 compared to high-density mature neutrophils (HDNs) [97,100]. CyTOF analysis by Shaul et al. revealed significant differences in the expression of CD10, CXCR4, CD94, and PD-L1 between LDNs and HDNs in advanced lung cancer patients [101]. Recently, a study by Valadez et al. reported overexpression of CD36, CD41, CD61 and CD226 on the LDNs of NSCLC patients [102]. Regarding their functionality, HDNs are represented by an antitumor phenotype while LDNs showed immunosuppressive effects on T cell proliferation, activation, and function [97,98]. It is suggested that the immature LDN subset represents G-MDSCs, known to be elevated in the blood of late-stage cancer patients with a suppressive phenotype [103]. The lectin-type oxidized LDL receptor 1 (LOX-1) was identified as a surface marker exclusively expressed on LDNs/G-MDSCs, but not on HDNs in both the peripheral blood and tumors of cancer patients; in contrast, in tumor-bearing mice LOX-1 was not associated with LDNs/G-MDSC [104]. Another study identified a mature CD10^+^ LDN subset with suppressive properties in healthy human donors receiving G-CSF treatment, thus addressing a TME-derived factor that potentially affects the generation and accumulation of LDN [96]. In the same study, CD10 was used to discriminate mature CD10^+^ from immature CD10^−^ neutrophils in the LDN fraction of cancer patients’ blood, presenting a promising opportunity for selective functional analysis of mature and immature LDN subsets. Defective expression of chemokine receptors CXCR1 and CXCR2 on the surface of LDNs was linked to a reduced in vitro migratory potential toward tumor-conditioned media in migration assays with LDNs from cancer patients and tumor-bearing mice [97,98]. In mouse models, TGF-β was reported to be the key driver of the HDN to LDN transition [105]. The identification of similar TME-derived factors that orchestrate the transition from regular neutrophils to LDNs in the circulation of cancer patients is a challenge that future investigation needs to focus on.

### 6.4. APC-Like Hybrid TANs

A study by Vono et al. suggested that freshly isolated human neutrophils can present antigens to autologous antigen-specific CD4^+^ T cells in a major histocompatibility complex class II (MHC-II; HLA-DR)-dependent manner [40]. More recently, a unique subpopulation of HLA-DR^+^ TANs with antitumor properties was reported in early-stage human lung cancer [106]. This population displayed characteristics of both granulocytes and APC-like dendritic cells and macrophages, and successfully induced tumor antigen-specific and nonspecific T cell responses. These so-called “hybrid TANs” decrease in numbers in large tumors, seemingly due to the TME-associated hypoxia. In terms of morphological characterization, hybrid TANs were found to have banded nuclei, indicating a possibility that they might derive from immature neutrophils under the impact of TME-secreted inflammatory factors such as GM-CSF and IFN-γ [42]. However, the presence of those hybrid TANs remains to be confirmed in vivo by more studies. The role of the TME secretome in both neutrophil polarization and unique subset generation during cancer progression needs to be further explored.

## 7. Pharmacological Targeting of the TME in the Context of Precision Medicine

### 7.1. Effects of Tumor-Infiltrating Myeloid Cells on Established Anticancer Therapies

In an established TME, tumor-infiltrating myeloid cells present numerous responses. More specifically, myeloid cells are responsible for clearing dead tumor cells and orchestrating the immune response following treatment-induced cancer regression. Myeloid cell populations interact with every type of anticancer therapy as shown in several experimental studies. These complex interactions between myeloid cells and cancer treatments can largely impact treatment outcome.

#### 7.1.1. Radio- and Chemotherapy

Cytotoxic therapies (radiation therapy and chemotherapy) remain the primary clinical approaches for many cancer types despite their sometimes limited efficacy. There is a dual interplay between cytotoxic therapies where myeloid cells can regulate treatment efficacy, but cytotoxic therapies can also regulate myeloid cell infiltration. Depending on the dose, its fractionation and the specific cancer type, radiotherapy (RT) can recruit myeloid cells and polarize them toward immunosuppressive phenotypes as reviewed by Vatner and Formenti [107]. Moreover, several experimental studies showed that depletion of TAMs, MDSCs and neutrophils enhance RT efficacy [108,109]. However, local irradiation with low-dose ionizing radiation in a transgenic mouse model of pancreatic cancer was shown to stimulate iNOS^+^ TAM accumulation. iNOS^+^ TAMs can promote T cell influx and thus improve tumor control and mouse survival [110]. TAMs and TANs can also play a role during chemotherapy (CT). ROS production by TAMs and TANs that facilitates the stimulation of tumor cell death has been observed upon treatment with oxaliplatin [111]. Moreover, interfering with CCR2^+^ TAMs and CXCR2^+^ TANs has shown significant effects when used along with CT [112,113]. On the other hand, TAMs and MDSCs can induce drug resistance through the production of cysteine cathepsins, which protect tumor cells from chemotherapeutic agents such as Taxol and even support tumor growth by promoting chronic inflammation [114,115].

#### 7.1.2. Targeted Therapy

Targeted cancer therapy takes advantage of the progress made in molecular profiling of tumors with known mechanisms of disease progression or treatment resistance, as well as the heterogeneity between primary and metastatic tumors and the dynamic changes of tumor profiles over time, as previously reviewed by others [116,117]. Targeted therapy uses molecules targeting specific enzymes, growth factor receptors and signal transducers to interfere with oncogenic mechanisms. Such drugs can drastically shrink tumors, but usually show short-lasting effects. There is therefore a need to examine the underlying tumor resistance mechanisms linked to the interactions between myeloid cells and targeted therapy. Small-molecule inhibitors have been clinically tested to target proteins with kinase activity such as BRAF, MEK, and MET [118,119]. Mutations of BRAF and MEK have been linked to oncogenesis. The development of resistance to BRAF inhibitors in a mouse melanoma model was associated with the restoration of the MDSC compartment. Depleting MDSCs by using antibodies (anti-Gr-1) or blocking their recruitment (CCR2 antagonist) did not allow the growth of BRAF-resistant melanoma tumors [120]. MET (hepatocyte growth factor receptor) is a target in several cancers, including non-small cell lung cancer, gastrointestinal cancer, and hepatocellular carcinoma [119]. Inhibition of MET affects neutrophils, since they depend on MET activity to infiltrate tumors and exert cytotoxic activities such as iNOS-mediated release of NO [121]. More recently, Haas et al. showed that in both melanoma patients and mice, tumors that acquired resistance to MAPK targeted therapy were represented by an immune-evasive TME with reduced and functionally impaired CD103^+^ DCs [122]. Alternatively, some targeted therapies also positively affect myeloid cells. MEK and BRAF inhibitors reverted BRAF-mutated melanoma-induced DC suppression in vitro [123].

#### 7.1.3. Immunotherapy

The T cell-mediated adaptive immune response against cancer is a complex, multistep process. At each step of this process, regulatory signals are essential to contain the destructive capacity of the adaptive immune system and prevent autoimmune toxicity. Co-signals are required for the activation of dendritic cells at the tumor site and of T cells in lymphoid organs. Tregs, MDSCs and metabolites in the TME can suppress the activity of T cells [124,125]. Tumors can thus take advantage of innate regulatory signals to evade or blunt the adaptive immune response. APCs migrate to the lymph node and present tumor antigens to T cells by interacting with the T cell receptor (TCR) via the major histocompatibility complex 1 (MHC-I), with the scope of stimulating tumor-specific T cell activation. However, the TCR-MHC-I interaction alone is not sufficient for T cell activation. The B7.1 (CD80) or B7.2 (CD86) molecules on the APC surface must bind CD28 on the T cell surface to provide a costimulatory signal for T cell activation [126]. CTLA-4 is a T cell surface receptor that competes with CD28 for B7, thus inhibiting this costimulatory signal. It also downregulates helper T cell activity and enhances Treg immunosuppressive activity [127]. The blockade of CTLA-4 was therefore considered a promising mechanism for anticancer immune activity. While CTLA-4 functions primarily in the lymph node, the other major checkpoint molecule that has been targeted operates in the TME. PD-1 is a transmembrane protein expressed on the surface of activated T cells as well as B cells, NK cells, T cells, and APCs [128]. It interacts with two main ligands: (i) PD-L2, primarily present on immune cells, and (ii) PD-L1, which has broad tissue expression, including on the surface of tumor cells [129,130]. The interaction of PD-1 with PD-L1 inhibits T cell receptor-mediated lymphocyte proliferation, cytokine secretion, and overall effector T cell function [131]. Antibodies blocking the PD-1/PD-L1 interaction led to increased effector T cell function in melanoma models, forming the basis for PD-1 and PD-L1 antibodies in cancer immunotherapy. Pivotal studies in melanoma led to the first FDA approval of PD-1 blockade agents in 2014 [132,133]. During the last few years, there has been a vast increase in clinical trials featuring PD-1 and PD-L1 blockade for cancer therapy. As thoroughly reviewed by Thomey and Zhang [134], the FDA has so far approved three anti-PD-1 antibodies: pembrolizumab (Keytruda), nivolumab (Opdivo), and cemiplimab (Libtay), and three anti-PD-L1 antibodies: atezolizumab (Tecentriq), durvalumab (Imfinzi), and avelumab (Bavencio). In addition, another anti-PD-1 antibody, dostarlimab (Jemperli), received FDA approval in August 2021 for the treatment of adult patients with mismatch repair-deficient (dMMR) recurrent or advanced solid tumors [135]. These immunothrapeutics have become the standard of care for several cancer types, including primary and metastatic lung, head and neck, gastric, colorectal and breast cancer, hepatocellular and renal cell carcinoma, Hodgkin’s and B cell lymphoma, and melanoma.

A high number of patients do not initially respond or become resistant along the course of immunotherapy [136]. The density and diversity of tumor-infiltrating immune cells are closely related to prognosis and prediction of treatment efficacy. Mapping the composition of immune infiltrates and their functional state within the TME is important in terms of both diagnosis and designing treatment strategies [137]. The TME can, in a simplified manner, be characterized as cold (non T cell inflamed) or hot (T cell inflamed), which is largely attributed to the levels of proinflammatory cytokine production and CD8^+^ T cell infiltration [138]. The so-called hot tumors are characterized by T cell infiltration and molecular signatures of immune activation, whereas cold tumors show striking features of T cell absence or exclusion. Hot tumors present higher response rates to immunotherapy, such as PD-L1/PD-1 therapy [139]. Therefore, various studies have focused on converting non-inflamed cold tumors into hot ones to achieve a better response to immunotherapy [140]. Another leading hypothesis to explain treatment failure is that tumor-infiltrating myeloid cells are responsible for resistance to immune checkpoint blockade. This hypothesis is supported by the fact that tumors largely infiltrated by immunosuppressive myeloid cells correlate with poor prognosis and immune checkpoint therapy resistance, with MDSCs and TAMs being implicated in this process [141,142]. MDSC depletion in experimental models was shown to enhance antitumor immune responses and help overcome resistance [143], while functional modulation of MDSCs by epigenetic drugs sensitized resistant experimental cancer models to immune checkpoint therapy [144,145]. TAMs can inhibit PD-1:PD-L1 therapy by internalizing anti-PD-1 mAbs via the Fc domain of the antibody and FcγRs expression by macrophages [146]. In addition, metabolic and inflammatory pathways were found to stimulate the expression of PD-L1 on myeloid cells [147,148]. Apart from the ability of immunosuppressive myeloid cells to inhibit immune checkpoint therapy, immune-stimulatory myeloid cells have proven vital for treatment success. Binding of monocytes and DCs by anti-PD-L1 mAbs was found to be partly responsible for controlling tumor growth [149,150]. Therefore, we can conclude that in the context of precision medicine, the identification and characterization of myeloid cell subpopulations and the factors that drive their polarization and recruitment in the TME are essential for providing combinatorial targets that will support a successful anticancer therapy.

### 7.2. Tumor-Infiltrating Cells as Targets of Complementary Therapies

Over the last few years, an emerging number of experimental and clinical studies have been published regarding approaches to target myeloid cell populations with protumor properties such as M2-like TAMs, N2-like TANs and MDSCs as summarized in Table 1. Immunosuppressive leukocytes can be targeted either by reducing their numbers through direct depletion or by blocking their recruitment into the TME and redirecting their functional polarization. Simultaneously, there have been attempts to enhance the recruitment and function of antitumor leukocytes. Depletion of myeloid cells can be achieved by using antibodies against myeloid cell-specific surface markers such as CD11b, Gr-1 or Ly6G in mice. Moreover, transgenic mouse strains with permanent or conditional myeloid cell ablation have been established. Experimental approaches such as these have proven that depletion of immunosuppressive myeloid cells can delay tumor growth, while the depletion of stimulatory myeloid cells has the opposite effect [86,151]. A major drawback for such approaches is the fact that myeloid cell depletion is not restricted to the TME, and a complete depletion strategy is not applicable in a human setting. Thus, approaches that interfere with the accumulation of suppressive myeloid cells in the TME appear to be more suitable for clinical praxis. Recruitment of TAMs in the TME relies on the CCL2:CCR2 and M-CSF:M-CSFR axis. Several mAbs, small-molecule inhibitors and RNA interference have been used to block these signaling pathways [152,153,154,155,156,157]. MDSCs can also act on CCL2 through their expression of CCR2 [158]. Another strategy of reducing protumor leukocyte accumulation in the TME suggests interfering with VEGF:VEGFR signaling. VEGFR is highly expressed by tumor-infiltrating leukocytes, especially immunosuppressive Tregs [159], and its interaction with VEGF is needed for their migration and polarization. Therefore, it is proposed that interfering with the VEGF:VEGFR axis can reduce Treg numbers and at the same time re-educate them toward stimulatory phenotypes [160]. Blockade of VEGF:VEGFR signaling was shown to reduce the recruitment of MDSCs into the TME of non-small cell lung carcinoma in a CCR2-dependent way [161]. Several anti-VEGF-based drugs, such as bevacizumab, avastin and axitinib, targeting VEGFR, are now approved in various cancer types [162,163,164]. Blockade of GM-CSF was shown to reduce monocytes and myeloid precursor cells and to result in delayed tumor progression. Opposingly, administration of GM-CSF to tumors was shown to induce tumor-specific T cells, probably as a result of DC activation and in addition to reduce the number of MDSCs [165,166]. Overall, the data so far support the idea that shifting the balance between the number of tumor-infiltrating leukocytes with pro- and antitumor functions is a strong treatment strategy that needs to be approached with caution with regard to interference with pathways involved in myelopoiesis.

Myeloid cells are characterized by high phenotypic plasticity that either promotes or inhibits their protumor properties. Transcriptional networks control the phenotype and function of myeloid cells, bringing the idea of reprogramming them into focus. Zoglmeier et al. showed that treatment of tumor-bearing mice with TLR3 or TLR9 agonists limited MDSC-mediated T cell suppression [167]. Additionally, in the same study, activation of the TLR9 ligand CpG stimulated MDSCs to produce Th1-activating cytokines and be differentiated into M1 TAMs in an IFN-α driven way. To functionally reprogram myeloid cells, cytokines can also be used. Delivery of IL-12 to the TME was linked to antitumor effects, correlating with the reprogramming of MDSCs, Tumor-associated dendritic cells (TADCs) and TAMs into antigen-presenting cells with CD8^+^ T cell activating potential [168,169]. The antitumor stimulating properties of IL-12 were confirmed by the clinical responses observed in patients with renal cell carcinoma, melanoma and peritoneal metastasis from ovarian cancer upon IL-12 treatment [170,171,172,173]. Treatment with human recombinant IL-12 was also shown to trigger transient changes in neutrophils, platelets, reticulocytes, lymphocytes, natural killer cells and CD34^+^ hematopoietic progenitor cells in healthy subjects [174]. Type I IFNs, particularly IFN-α and IFN-β, strongly induce myeloid cell polarization, mostly affecting MDSCs and TADCs [175,176]. Although IFN-α has shown promising results in hematopoietic cancers, there has not been much success in solid tumors [177]. Preclinical studies that combined IFN-β stimulation of myeloid cells with simultaneous blockade of TGF-β signaling presented a solid approach of reprogramming MDSCs and DCs in support of CD8^+^ T cell responses [178].

Several strategies to down- or upregulate myeloid cell transcription factors such as STAT3 and NF-kB have also been attempted [179,180]. STAT3 is regarded as a leading stimulator of the immunosuppressive activity of myeloid cells [181,182]. Upon selective delivery of STAT3 inhibiting small interfering RNA (siRNA), TLR9-expressing myeloid cells such as G-MDSCs displayed reduced immunosuppressive capacity [183,184,185,186]. Treatment with a novel small-molecule STAT3 inhibitor blocked hepatocellular carcinoma tumor growth in mouse studies [187] and is currently being tested in clinical trials. On an epigenetic level, the use of histone deacetylase inhibitors (HDACi) that interfere with chromatin remodeling affected MDSC numbers and function. Inhibition of HDAC reduced the number of MDSCs as well as the expression of enzymes, including ARG-1 and iNOS, sensitizing several experimental cancer models to immune checkpoint therapy [144,145]. HDACi are currently studied in combination with checkpoint blockers in patients with metastatic and unresectable HER2/neu-negative breast and other cancers [148,188].

IDO, ARG-1, iNOS and COX2 are enzymes expressed by tumor-infiltrating myeloid cells, which have been under investigation since they are considered to be strongly connected to immunosuppressive functions. IDO is a tryptophan degrading enzyme induced in MDSCs, TADCs, TAMs, and to some extent in TANs in response to proinflammatory cytokines, TLR ligands, hormones, PGE2, and contact-dependent interactions such as the B7:cytotoxic T lymphocyte antigen 4 (CTLA-4) axis [189,190,191]. IDO mediates the conversion of tryptophan to kynurenine, which affects the induction of T cell anergy, apoptosis and commitment of CD4^+^ T cells toward immunosuppressive Tregs [192]. These Tregs can recruit and activate MDSCs, which in turn suppress T cells [193]. IDO expression correlates with decreased survival and increased risk of metastasis in several cancers [194]. In animal studies, genetic inhibition of IDO led to protumor granulocyte infiltration and activation in the TME [195]. Several IDO inhibitors are currently clinically validated either as mono- or combinatorial therapy [196]. ARG-1 converts L-arginine into L-ornithine and urea, and its expression is often upregulated in MDSCs, Μ2 TAMs and tolerogenic dendritic cells (tolDCs) under the influence of factors such as PGE2, GM-CSF, TGF-β, IL-6 and IL-10 [197,198,199,200]. Expression of ARG-1 by myeloid cells has been linked to protumor properties [201,202,203]. iNOS is also an L-arginine converting enzyme, with NO and citrulline being the downstream products. iNOS is expressed by M1 TAMs, inflammatory DCs and MDSCs as a response to IL-1β, IL-6, IFN-γ, TNF-α and TLR4 agonists. iNOS and NO have been linked to both anti- and protumor activities influenced by the TME, the genetic background and the cell type [204,205]. Inhibitors such as nor-*N*-hydroxy-L-arginine and amino-guanidine successfully downregulate ARG-1 and iNOS expression in MDSCs, restoring T cell function and delaying tumor progression [206]. More recent studies have also shown promising results in a combination of ARG-1 inhibition with immune checkpoint therapy or RT [193,207].

## 8. Outlook—Future Directions

With this review we aimed to document the complexity of the interactions between myeloid cells and the TME, with a primary focus on neutrophils. Neutrophils are a highly heterogeneous population with reported phenotypic and functional plasticity. Tumor-associated neutrophils display opposing roles during cancer progression. Numerous TME-derived factors take part in an interplay that orchestrates neutrophil release, recruitment, and functional polarization. Simultaneously, neutrophils are active stimulators of the TME, by secreting factors that shape immune interactions but also driving a feedback loop that affects their own fate. Immunotherapies are of key importance in several cancer types and stages. As shown in this review, successful immunotherapies rely on a coordinated targeting of leukocytes to harness their immune-stimulatory potential and limit their suppressive properties. Thus far, in the context of precision medicine, efforts have been invested in the development of pharmacological compounds that either alter cell numbers or repolarize their functions. Such strategies have resulted in significant data in several preclinical models. Many of these compounds are currently validated in preclinical and clinical trials. What has been made evident in this review is that targeting neutrophils is still under-represented in clinical studies, and that neutrophils are most of the time considered part of the MDSC fraction. This fact derives from the lack of comprehensive neutrophil subset characterization that would allow researchers to target them more precisely. The need for neutrophil surface markers that would identify distinct TAN subpopulations or indicate polarization states of both TAN and peripheral blood neutrophils are evident. Simultaneously, a functional characterization of TANs with pro- and antitumor phenotypes and the identification of the major TME-derived factors of neutrophil polarization would be of vital significance to exploit the full potential of neutrophils as complementary targets in anticancer precision therapies.

## Figures and Tables

**Table 1:** Pharmacological compounds targeting TME-derived factors that affect neutrophil function, currently in clinical trials. Data collected on ClinicalTrials.gov.

Target	Type	Name	Effect on Neutrophils/MDSCs
VEGFR	Blocking mAb	bevacizumab	Reduces MDSC recruitment into TME
	Blocking mAb	axitinib	
	c-Met and VEGFR2 inhibitor	cabozantinib	
GM-CSF	Oncolytic virus	OncoVEX^GM-CSF^	Reduces monocyte and myeloid precursor cell numbers
	Recombinant human GM-CSF	sargramostim	
TLR9	TLR9 agonist	CMP-001	Stimulates Th1-activating cytokine production by MDSCs
	TLR9 agonist	SD-101	
IL-12	Recombinant human IL-12	rHuIL-12	Reprograms MDSCs into APCs
	IL-12 gene therapy activator	veledimex	
IFN-α	Pegylated IFN-a	pegasys	Stimulates MDSC polarization
IFN-β	Oncolytic Virus	VSV-IFNβ-NIS	Stimulates MDSC polarization
IFN-β, TGF-β	mRNA	Fβ2 fusokine	Reprograms MDSCs in favor of CD8+ T cell responses
STAT3	STAT3 inhibitor	TTI-101	Reduces immunosuppressive capacity of G-MDSCs
	STAT3 inhibitor	napabucasin	
	STAT3 inhibitor	pyrimethamine	
IDO1	IDO inhibitor	indoximod	Affects protumor granulocyte infiltration in the TME
	IDO inhibitor	epacadostat	
	IDO/TDO inhibitor	linrodostat	
ARG-1	Recombinant human Arg1	pegzilarginase	Expression by myeloid cells is linked to protumor properties
	Arg1 inhibitor	INCB001158	
HDAC	HDAC inhibitor	panobinostat	Reduces MDSCs and their expression of ARG-1 and iNOS
	HDAC inhibitor	belinostat	
